# The relaxant effect induced by *Allium sativum* L. bulb aqueous extract on rat isolated trachea

**DOI:** 10.4103/0973-1296.75879

**Published:** 2011

**Authors:** Badreddine Fehri, Mueen K.K. Ahmed, Jean-Marc Aiache

**Affiliations:** *Department of Pharmacology and Toxicology, Tunisia Pharmaceutical Industries Company (SIPHAT), Fondouk Choucha-Radès, 2013 Ben Arous, Tunisia*; 1 *Department of Pharmaceutical Sciences, College of Clinical Pharmacy, King Faisal University, P. O. Box 400, Al-Ahsa-31982, Kingdom of Saudi Arabia*; 2 *Department of Biopharmaceutics, Faculty of Pharmacy, Auvergne University, 28, Place Henri Dunant - BP 38, 63001 Clermont-Ferrand, France*

**Keywords:** *Allium sativum* L. bulb aqueous extract, rat isolated trachea

## Abstract

**Background:**

Garlic plays an important role in complementary and alternative medicine. Most people believe in and use herbal products even when they have not been as thoroughly researched as garlic. Garlic is also known for its beneficial effects on the cardiovascular system.

**Materials and Methods:**

The relaxant effect of *Allium sativum* L. bulb aqueous extract (ASBAE) containing 0.06%-0.10% of allicin was studied on isolated smooth muscle of trachea of rats precontracted using acetylcholine (10^−5^ M).

**Results:**

It was found that ASBAE induced a dose-dependent relaxation with recorded EC _50_ values of 71.87 ± 5.90 *µ*g/mL (n = 7). Pretreatments with mepyramine (10^−7^ M), methysergide (10^−7^ M), caffeine (10^−6^ M), theophylline (10^−6^ M), nifedipine (10^−6^ M), and dipyridamole (10^−6^ M) did not alter ASBAE concentration-response curves. In turn, concentration-response curves to ASBAE were significantly shifted toward right in the presence of aspirin (3.10^−3^ M), indomethacin (10^−6^ M), prazosin (10^−6^ M), and propranolol (10^−7^ M).

**Conclusion:**

It is suggested that the recorded relaxation results are due to the release of prostaglandins E _1_ and E _2_ consecutively to α- and β-adrenoreceptor stimulation.

## INTRODUCTION

*Allium sativum*, commonly known as garlic, is one of the species belonging the onion family Alliaceae. Garlic is largely used around the world for its pungent flavor as a seasoning or condiment.[1] It is a fundamental component in many or most dishes of various regions, including eastern Asia, south Asia, Southeast Asia, the Middle East, northern Africa, southern Europe, and parts of South and Central America.[2] It contains the sulfur-containing compounds alliin, ajoene, diallylsulfide, dithiin, S-allyl cysteine and also enzymes, vitamin B, proteins, minerals, saponins, flavonoids, and maillard reaction products, which are non-sulfur-containing compounds. Furthermore, a phytoalexin called allixin (3-hydroxy-5-methoxy-6-methyl-2-penthyl-4H-pyran-4-one) was found, a nonsulfur compound with a γ-pyrone skeleton structure with antioxidative effects, antimicrobial effects, antitumor promoting effects, inhibition of aflatoxin B_2_ DNA binding, and neurotrophic effects.[3–8]

*A. sativum* L. was also demonstrated to reduce cardiovascular risk factors, especially blood pressure,[9–11] hyperlipidema, and atherosclerosis.[12] Garlic is also shown to have significant relaxant effects on the airway smooth muscles. In this regard, it has been observed that administering garlic prevented the occurrence of high altitude breathing symptoms.[13] In 1998, Fallon *et al*.[14] demonstrated that active substances that are found in garlic caused vasorelaxation in the pulmonary arterial segments. Recently, a report indicated that a mixture of herbs containing *A. sativum* L. reduced an elevated respiratory rate in horses with recurrent airway obstruction.[15] In contrast, the mechanism of action of *A. sativum* L. in airway smooth muscle remains unclear. Hence, the present study is to investigate and identify the possible mechanism for the relaxant effects caused by *A. sativum* L. extracts.

## MATERIALS AND METHODS

### Plant material

The drug studied was a *A. sativum* L. bulb aqueous extract (ASBAE) obtained from Finzelberg Laboratories (Germany), which answered the following parameters:

- Ratio of dried plant/extract: 6:1
- Allicin content was determined using gas chromatography: 0.10%

### Drugs used

ASBAE (Finzelberg), acetylcholine HCl, propranolol HCl, dipyridamole were purchased from Sigma, St. Louis, MO, USA, nifedipine from Dolder, Basel, Switzerland, aspirin DL-lysine (DL-lysine acetylsalicylate) from Sanofi-aventis Paris, France, prazosin HCl from Pfizer Kalamazoo, USA, caffeine, theophylline sodium anisate, methysergide maleate, indomethacin, and mepyramine maleate from Siphat, Fondouk Choucha-Radès, Tunisia.

Prior to their dilution in Tyrode’s solution, indomethacin, dipyridamole, and nifedipine were dissolved in absolute ethanol and then diluted in Tyrode’s solution. Theophylline and aspirin were used from ampoules for injection (Siphat, Fondouk Choucha-Radès, Tunisia and Sanofi-aventis, Paris, France). The other substances were directly dissolved in Tyrode’s solution. The required concentrations of the compounds were added to the organ bath so that the volume of ethanol never exceeded 0.05 mL (0.4% by volume). Such quantity of ethanol had no effect on acetylcholine and ASBAE responses on isolated rat trachea.

### Rat trachea *in vitro*

Male rats weighing 300-400 g were selected in a homogeneous breeding center (Central Animal House, Tunisia Pharmaceutical Industries Company, Fondouk Choucha-Radès, Tunisia). They were sacrificed by a blow and exsanguinated. The trachea was removed immediately and cut into small segments of 3-4 rings, which were equilibrated under a resting tension of 1.5 g and suspended in 10 mL baths containing Tyrode’s solution at 37°C ± 0.5°C and the tissue was aerated continuously with 95% O_2_ and 5% CO_2_. The composition of the Tyrode’s solution was (mM): [NaCl: 139.2; KCl: 2.7; CaCl_2_ : 1.8; MgCl_2_ : 0.49; NaHCO_3_ : 11.3; Na_2_HPO_4_ : 0.4; glucose: 5.5]. Tension was measured isometrically with Ugo Basil strain gauges displayed on Geminy Ugo Basile recorders (Italy).

### Experimental procedure

The tracheal rings (3 to 4) were placed in organ bath for 85 min and washed with Tyrode solution at every 15-min time interval. The tissue was contracted using acetylcholine (ACh 3.10^−3^ M) until maximal contraction was achieved. During that stage, the tissue was subjected to relaxation using with theophylline (THEO 3.10^−3^ M), further washed repeatedly and allowed to rest until it returned to baseline tension. When baseline tension was reached, it was again contracted with ACh 10^−5^ M, and maximal tension was obtained, during this time, it was studied to establish concentration-response curves to ASBAE by the method of cumulative addition. The final addition was followed by addition of THEO 3.10^−3^ M to obtain complete relaxation. Pretreatments with drugs were performed 30 min before ACh 10^−5^ M contractions. The relaxations induced by ASBAE were expressed as a percentage of relaxation vs complete relaxation (E_MAX_). The −log EC_50_ (pD_2_) values, defined as the negative log concentration-effect curves, were evaluated graphically from each experiment.

### Statistical analysis

All data were expressed as mean ± SEM. Data of relaxant effects of different concentrations of extracts were compared with the results of negative and positive control using Student *t* test. Significant differences with controls are shown as *P < 0.05 and ***P < 0.001.

## RESULTS

**Figure 1:**
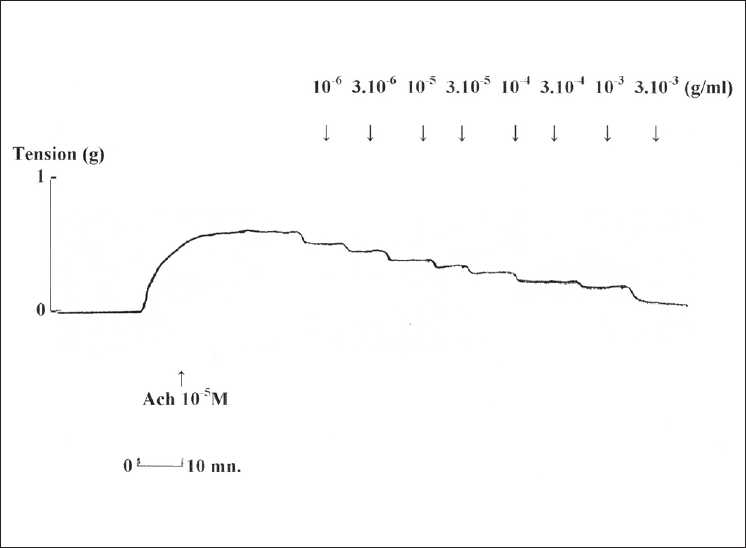
Representative trace from acetylcholine (ACh 10−^5^ M) precontracted rat isolated trachea showing cumulative dose-response to *Allium sativum* L. aqueous extract. Concentrations (10−^6^ to 3.10−^3^) are in g/mL

ASBAE exhibited a dose-dependent relaxant effect on the rat isolated trachea precontracted with ACh 10^−5^ M [Figure 1]. On basal tone of isolated tissue, ASBAE showed mild contractile effects at lower doses ranging from 10^−6^ to 10^−4^ g/mL, followed by relaxant effects at higher doses (from 10^−3^ to 3.10^−3^ g/mL) (data not shown).

**Figure 2:**
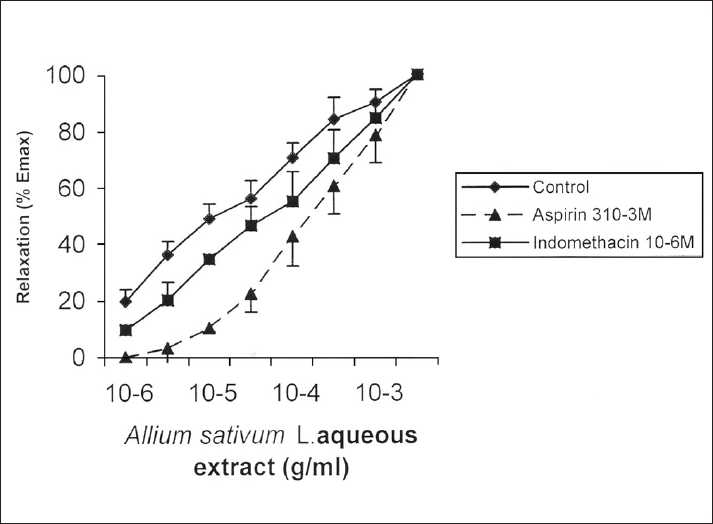
Cumulative concentration-response curves for *Allium sativum* L. extract on rat isolated trachea. Values are mean ± SEM

**Figure 3:**
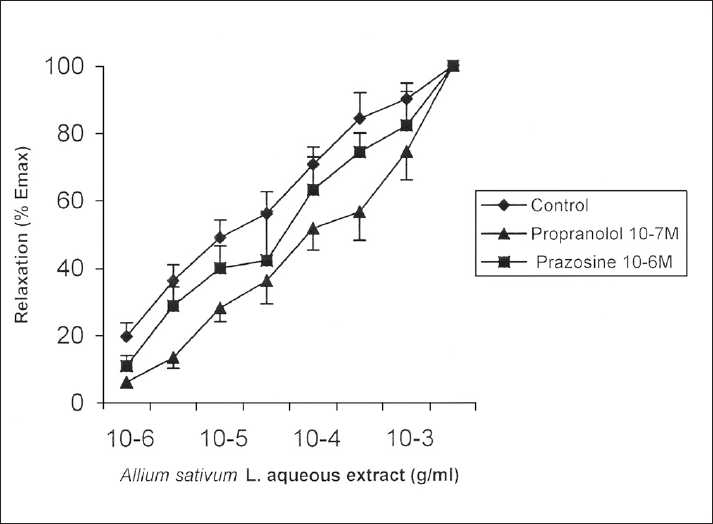
Cumulative concentration curves for *Allium sativum* L. extract on rat isolated trachea. Values are mean ± SEM

Pretreatments with mepyramine 10^−7^ M and methysergide 10^−7^ M, ASBAE concentration-response curves were not significantly modified as observed by pD_2_ values [Table 1]. Statistical analysis of pD_2_ values also failed to detect any difference in the relaxant effect of ASBAE between control and tracheal rings treated with caffeine (10^−6^ M), theophylline (3.10^−6^ M), nifedipine (10^−6^ M), and dipyridamole (10^−6^ M) [Table 1]. In turn, pretreatments with aspirin (3.10^−3^ M) and indomethacin (10^−6^ M) caused right shift to ASBAE concentration-response curves, and pD_2_ values significantly decreased [Table 1 and Figure 2]. Prazosin (10^−6^ M) and propranolol (10^−7^ M) had the same effect (*P* < 0.05 and *P* < 0.001) [Table 1 and Figure 3].

**Table 1:** pD_2_ (–log EC_50_) of ASBAE and its influence of various pretreatments on relaxant effect

Pretreatment (M)	N	pD_2_
Control	7	4.14 ± 0.03
Caffeine (10^−6^)	3	4.17 ± 0.06
Theophylline (3.10^−6^)	3	4.18 ± 0.03
Mepyramine (10^−7^)	3	4.27 ± 0.15
Methysergide (10^−7^)	3	4.31 ± 0.10
Dipyridamole (10^−6^)	3	4.28 ± 0.08
Nifedipine (10^−6^)	3	4.48 ± 0.25
Aspirin (3.10^−3^)	3	3.84 ± 0.09*
Indomethacin (10^−6^)	3	4.01 ± 0.01*
Prazosin (10^−6^)	3	4.03 ± 0.01*
Propranolol (10^−7^)	3	3.25 ± 0.13***

ASBAE, *Allium sativum* L. bulb aqueous extract, Values are means ± SEM. The relaxations induced by ASBAE were expressed as a percentage of maximal relaxation (E_MAX_). The pD_2_ (–log EC_50_) values were derived graphically from the log concentration-effect curves; n indicates the number of tracheal preparations. Significant differences with controls are shown as:

* *P* <0.05

*** *P* < 0.001

## DISCUSSION

The present work indicated that ASBAE induced multiple effects on rat isolated trachea according to the level on tone. On basal tone trachea, ASBAE induced contractile effects at low doses (10^−6^ to 10^−4^ g/mL), whereas at higher doses (10^−3^ to 3.10^−3^ g/mL), it induced relaxation effect.

Furthermore, it was also demonstrated through the present work that ASBAE induced a dose-dependent relaxation with EC_50_ values of 71.87 ± 5.90 µg/mL (n = 7) on the rat isolated trachea precontracted with ACh 10^−5^ M. In the presence of both, mepyramine 10^−7^ M, an antagonist of H_1_ receptors[16] and methysergide 10^−7^ M, an antagonist of serotonin (5-Hydroxytryptamine) receptors,[17] the induced relaxation of ASBAE was not significantly modified. The present study suggests that ASBAE-induced relaxation may not be resulting from interactions with histamine and serotonin receptors. In the other hand, caffeine (10^−6^ M) and theophylline (3.10^−6^ M), two phosphodiesterase (PDE) inhibitors,[18] and nifedipine (10^−6^ M) and dipyridamole (10^−6^ M), two compounds acting as calcium antagonists,[19] did not alter ASBAE-induced relaxation; hence, it is possible to suggest that the recorded relaxation does not involve inhibition of cyclic nucleotide phosphodiesterase or a calcium blocking influx phenomenon.

The prostaglandin synthetase inhibitors, aspirin (3.10^−3^ M) and indomethacin (10^−6^ M),[19] significantly antagonized ASBAE-induced relaxation. The present outcome may possibly suggest that the recorded relaxation could be mediated due to the release of the relaxant metabolites of arachidonic acid (AA) produced under the activation of cyclooxygenase. It has been shown that garlic prevents platelet aggregation by selectively inhibiting platelet thromboxane (TX) synthesis and reducing vascular prostacyclin (PGI_2_).[20,21]

The lungs of many animal species, including man, are rich in prostaglandins (PGs) that do influence the tone of tracheal and bronchial muscles. Many studies demonstrated that PGs of the E series and PGs of F series, respectively, induce a relaxation and a contraction of tracheal smooth muscle *in vitro*. Takano *et al*.[22] particularly studied the effects of PGs E_1_, E_2_, F_2α_, A_1_, A_2_, and B_2_ on guinea pig-isolated trachea and showed that F_2α_, B_2_, and A_2_ produced contraction, whereas PGs E_1_ and E_2_ produced relaxation. The same authors reported that PG A_1_ produced no response. The same result was obtained by Coleman and Kennedy,[23] who studied the effect of 12 PGs on the guinea pig-isolated trachea. PGs of series E are considered to be bronchodilators not only in experimental animals but also in man.[24] Taking into consideration, based on these observations, it is now possible to suggest that active components contained in ASBAE induced the recorded relaxation by a stimulation of PGs E_1_ and E_2_, and further, the effect was antagonized by aspirin and indomethacin pretreatments.

It is also reported that PGs E_1_ and E_2_ cause relaxation when tone is present and contraction when tone is absent.[19,24,25] This may explain the contractile effects induced at low doses (from 10^−6^ to 10^−4^ g/mL) by ASBAE on basal tone trachea.[26] This contraction phase could therefore be mediated by PGs E_1_ and E_2_. When basal tone has reached a sufficient level, then a relaxation phase occurred (from 10^−3^ to 3.10^−3^ g/mL) probably due to the release of PGs E_1_ and E_2_.

Pretreatments with the respective α- and β-adrenoreceptor blockers, prazosin 10^−6^ M[27,28] and propranolol 10^−7^ M, caused right shift of ASBAE concentration-effect curves. The order of potency of the recorded antagonism was propranolol 10^−7^ M > prazosin 10^−6^ M [Table 1 and Figure 3].

This result may suggest that active components present in ASBAE could stimulate both α- and β-adrenergic receptors with a higher affinity for β than for α receptors. The affinity of various *Allium* species for adrenergic (α_1_, α_2_, β_1_, and β_2_) receptors has recently been studied. Nencini *et al*.[29] reported that *A. sativum* L. did not show high affinity for α_1_ and α_2_ receptors and showed a partial affinity for β_1_ receptors, whereas wild-type *Allium* species presented a greater affinity for these receptors, in particular for β_2_ receptors (*Allium neapolitanum* and *Allium subhirsutum* species).

## CONCLUSION

Further investigations need to be undertaken to determine the affinity of *A. sativum* L. for adrenergic receptors on rat trachea. The link between PGs E_1_ and E_2_ and α- and β-adrenergic receptor stimulation by which active components contained in ASBAE relax the rat isolated trachea should also be clarified. In this respect, it has been suggested that PGs E_2_ exert their biologic actions via at least 4 receptor subtypes (EP_1_ -EP_4_) and that EP_1_ could act as a modulator of β_2_ -adrenergic receptor.[30] Active components present in ASBAE could possibly relax the rat isolated trachea by such mechanism of action.
